# How Does Digital Innovation Drive Corporate Sustainability? A Systematic Literature Review

**DOI:** 10.12688/f1000research.187511.1

**Published:** 2026-08-14

**Authors:** Joni Prasetiyanto, Mochammad Al Musadieq, Muhammad Faisal Riza, Benny Hutahayan

**Affiliations:** 1 Universitas Brawijaya Fakultas Ilmu Administrasi, Malang, East Java, Indonesia

**Keywords:** Digital Innovation; Corporate Sustainability; Esg Performance; Digital Transformation; Systematic Literature Review

## Abstract

This study systematically reviews recent empirical evidence on the relationship between digital innovation and corporate sustainability. Using the Scopus database, the review identified 231 articles through the search string (“digital” OR “digital innovation” OR “digitalization”) AND “corporate sustainability”. After applying inclusion criteria covering publications from 2023 to 2026, English-language journal articles, open access availability, and quantitative empirical design, 49 articles were retained for synthesis. The findings show that digital innovation is conceptualized through multiple forms, including digital transformation, digital finance, artificial intelligence, digital accounting, supply chain digitalization, digital leadership, executives’ digital attention, and digital–green integration. Most studies indicate that digital innovation positively contributes to ESG performance, corporate sustainability performance, sustainable development performance, and sustainability efficiency. However, the relationship is not automatic or universally linear. Its effect depends on mediating mechanisms such as green innovation, operational efficiency, financing access, information transparency, supply chain coordination, resource orchestration, and governance improvement. The review also identifies important boundary conditions, including digital maturity, organizational capability, leadership support, digital infrastructure, environmental regulation, and institutional quality. This study contributes by organizing fragmented empirical evidence into an integrated thematic framework and highlighting future research directions for global scholars examining digital transformation, ESG practices, and sustainability-oriented corporate strategy worldwide.

## 1. Introduction

Digital innovation has increasingly become a strategic driver of corporate transformation in the era of sustainability-oriented business competition. The rapid diffusion of digital technologies, including artificial intelligence, big data, blockchain, cloud computing, digital finance, robotic process automation, and digital platforms, has reshaped how firms organize resources, coordinate operations, disclose information, and interact with stakeholders. At the same time, corporations are under growing pressure to improve environmental, social, and governance performance as part of their long-term sustainability agenda. In this context, digital innovation is no longer viewed merely as a technological modernization tool, but as a strategic mechanism for strengthening corporate sustainability, improving ESG performance, and supporting sustainable development performance ( Ji et al., 2023; Li & Zhao, 2024; Pu, 2025; Sharma & Radhika, 2026; Ying & Jin, 2023).

Digital innovation refers to the creation, adoption, and strategic use of digital technologies to redesign business processes, products, services, organizational routines, and value creation mechanisms. In the context of corporate sustainability, digital innovation enables firms to improve the efficiency and accountability of their economic activities while responding to environmental and social pressures. Digital technologies allow firms to collect, process, and analyze large volumes of operational and stakeholder-related data, thereby improving decision-making quality, reducing information asymmetry, and enhancing the transparency of sustainability practices. Prior studies show that digital transformation improves corporate sustainability by strengthening operational efficiency, innovation capability, resource allocation, and disclosure quality ( Ji et al., 2023; Li & Zhao, 2024; Pu, 2025; Yao et al., 2025). Therefore, digital innovation can be understood not only as a technological change, but also as a strategic capability that enables firms to align economic performance with environmental and social responsibility.

Digitalization also helps firms overcome several constraints that often hinder sustainability-oriented investments. Corporate sustainability requires long-term commitments to green innovation, cleaner production, responsible supply chains, ESG reporting systems, and stakeholder engagement. However, these initiatives are frequently constrained by limited financial resources, weak information transparency, inefficient resource allocation, and organizational inertia. Digital finance, FinTech, artificial intelligence, and big data analytics can help firms reduce financing constraints, improve access to capital, optimize resource use, and strengthen monitoring systems ( Hu et al., 2023; Su & Liang, 2025; Wang & Zhang, 2025; Liu et al., 2025a). In addition, digital accounting, digital reporting, and information systems support sustainability by improving financial transparency, reporting accuracy, and accountability to stakeholders ( Al-Taani et al., 2024; Alshehadeh et al., 2024; Neiroukh & Çağlar, 2025; Novicka, 2025). These findings suggest that digitalization contributes to corporate sustainability through both operational and governance pathways.

Recent studies further emphasize that the sustainability value of digital innovation becomes stronger when digital initiatives are integrated with green transformation. Digital technologies alone may improve efficiency, but their contribution to sustainability becomes more substantial when they are directed toward green innovation, carbon reduction, resource efficiency, sustainable supply chain management, and ESG-oriented governance. Studies on digital–green fusion and digital and green transformation show that the integration of digital and environmental strategies produces stronger sustainability outcomes than isolated digital or green initiatives ( Yang & Luo, 2024; Zhu & Li, 2025). Similarly, research on supply chain digitalization and digital trade demonstrates that digital integration across suppliers, customers, and business partners improves sustainability through better traceability, coordination, financing efficiency, and human capital optimization ( Guo & Kuang, 2025; Li et al., 2025b; Zhang et al., 2025). Thus, digital innovation should be understood as a key enabler of corporate sustainability when it is embedded in broader organizational, supply chain, and institutional systems oriented toward long-term sustainable development.

Despite this growing body of evidence, the relationship between digital innovation and corporate sustainability remains theoretically and empirically fragmented. First, digital innovation has been conceptualized in multiple ways, including digital transformation, digital finance, artificial intelligence, digital accounting, digital trade, digital leadership, executives’ digital attention, government open data platforms, and digital–green fusion ( Al-Taani et al., 2024; Lulaj & Brajković, 2025; Xu & Xu, 2025; Yang & Luo, 2024; Zada et al., 2025). This conceptual diversity enriches the literature, but it also makes it difficult to develop a coherent understanding of how different forms of digital innovation contribute to sustainability outcomes. Second, although many studies report a positive effect of digital innovation on ESG performance or corporate sustainability, others show nonlinear, conditional, or insignificant effects. For instance, digital finance and digital maturity may initially impose adjustment costs before generating sustainability benefits, resulting in U-shaped relationships ( Hu et al., 2023; Yin et al., 2024; Yilmaz et al., 2025). In addition, some evidence suggests that digital transformation may not directly improve ESG ratings when governance, transparency, or institutional readiness is weak ( Samidi & Kwan, 2024).

The literature is also fragmented in explaining the mechanisms and boundary conditions of the digital innovation–sustainability relationship. Existing studies identify green innovation, digital green innovation, operational efficiency, information transparency, financing access, supply chain coordination, management efficiency, analyst attention, resource orchestration, and organizational change as important mechanisms linking digital innovation and sustainability ( Ibrahim et al., 2026; Liu et al., 2025b; Tan & Cui, 2026; Yao et al., 2025; Zhang et al., 2026a). At the same time, the strength of this relationship depends on boundary conditions such as digital maturity, organizational inertia, digital infrastructure, marketization level, environmental regulation, leadership support, board diversity, media attention, and institutional policy support ( Fan et al., 2025; Lyu et al., 2026; Sang et al., 2026; Xu & Xu, 2025; Zhu & Li, 2025). These findings indicate that digital innovation does not automatically produce sustainability outcomes. Rather, its impact depends on the organizational and institutional context in which it is implemented.

Therefore, a systematic literature review is needed to consolidate recent empirical evidence and clarify how digital innovation contributes to corporate sustainability. This study addresses this need by reviewing 49 quantitative empirical articles published between 2023 and 2026 and indexed in the Scopus database. By synthesizing these studies, this review aims to identify the dominant research streams, major empirical findings, key mechanisms, boundary conditions, and remaining research gaps in the relationship between digital innovation and corporate sustainability. This review contributes to the literature by organizing fragmented evidence into an integrated thematic structure and by showing that digital innovation should be understood not only as technology adoption, but also as a strategic, organizational, and institutional capability for sustainable corporate development.

Specifically, this study is guided by three research questions. First, what forms of digital innovation have been examined in relation to corporate sustainability? Second, through what mechanisms does digital innovation influence corporate sustainability outcomes? Third, what contextual factors strengthen, weaken, or complicate the relationship between digital innovation and corporate sustainability? By answering these questions, this review provides a structured understanding of the recent development of the field and offers future research directions for scholars interested in digital transformation, ESG performance, sustainable development, and corporate sustainability.

## 2. Method

### 2.1 Research design

This study employed a systematic literature review approach to identify, evaluate, and synthesize empirical studies examining the relationship between digital innovation and corporate sustainability. A systematic literature review was considered appropriate because it enables researchers to map existing knowledge, identify dominant research patterns, and reveal gaps for future inquiry in a transparent and replicable manner ( Tranfield et al., 2003; Snyder, 2019). The review process was guided by the Preferred Reporting Items for Systematic Reviews and Meta-Analyses (PRISMA) framework to ensure methodological rigor, transparency, and traceability in article selection ( Page et al., 2021).

### 2.2 Database and search strategy

The literature search was conducted using the Scopus database, which was selected because of its broad coverage of peer-reviewed international journals across the fields of business, management, innovation, sustainability, and information systems. The search focused on studies discussing digital-related innovation and corporate sustainability. The following search string was used:

### 2.3 Inclusion and exclusion criteria

The inclusion and exclusion criteria were established to ensure that only studies relevant to the research objective were retained. Articles were included if they met the following criteria: (1) published between 2023 and 2026; (2) written in English; (3) published as peer-reviewed journal articles; (4) available as open access; (5) explicitly discussed digital innovation, digitalization, or related digital transformation issues; (6) examined corporate sustainability or sustainability-related organizational outcomes; and (7) employed a quantitative research approach.

Articles were excluded if they were not directly related to digital innovation and corporate sustainability, were conceptual or purely qualitative in nature, were review papers, conference papers, book chapters, editorials, or notes, or did not provide sufficient empirical evidence for synthesis. After reading the abstracts and full texts, only quantitative empirical studies that directly aligned with the focus of this review were retained.

### 2.4 Screening and selection process

The article selection process was conducted in three stages. First, an identification stage was carried out through the Scopus database using the predefined search string, resulting in 231 articles. Second, a filtering stage was conducted by limiting the results to articles published from 2023 to 2026, written in English, categorized as journal articles, and available as open access. This process reduced the number of articles to 151. Third, the remaining articles were screened by reading their abstracts and full texts to assess their relevance to the topic and methodological suitability. At this stage, studies that did not use quantitative methods or did not directly examine the relationship between digital innovation-related constructs and corporate sustainability outcomes were excluded. As a result, 49 articles were selected as the final sample for review and synthesis.

The selection process can be summarized as follows in Table 1:

**Table 1.** Selection process.

Stage	Criteria Applied	Number of Articles
Initial search	Scopus search using the keywords: (“digital” OR “digital innovation” OR “digitalization”) AND “corporate sustainability”	231
Filtering	2023–2026, English, journal article, open access	151
Eligibility screening	Abstract and full-text reading; only relevant quantitative studies retained	49
Final articles reviewed	Articles included in the systematic literature review	49

Data were extracted systematically from the 49 selected articles. The extraction process focused on key bibliographic, methodological, and substantive information, including author name, year of publication, research context, country or region, industry sector, research objective, theoretical foundation, research variables, sample characteristics, data analysis technique, and main findings. Particular attention was given to how digital innovation was conceptualized and measured, as well as how corporate sustainability was operationalized, whether through environmental, social, economic, ESG, or broader sustainability performance indicators.

This extraction process enabled the study to classify the selected articles according to research themes, methodological approaches, theoretical perspectives, and empirical findings. The extracted information also served as the basis for identifying research trends, dominant variables, methodological tendencies, and gaps in the literature. Figure 1 shows the article selection process in detail.

**Figure 1.**
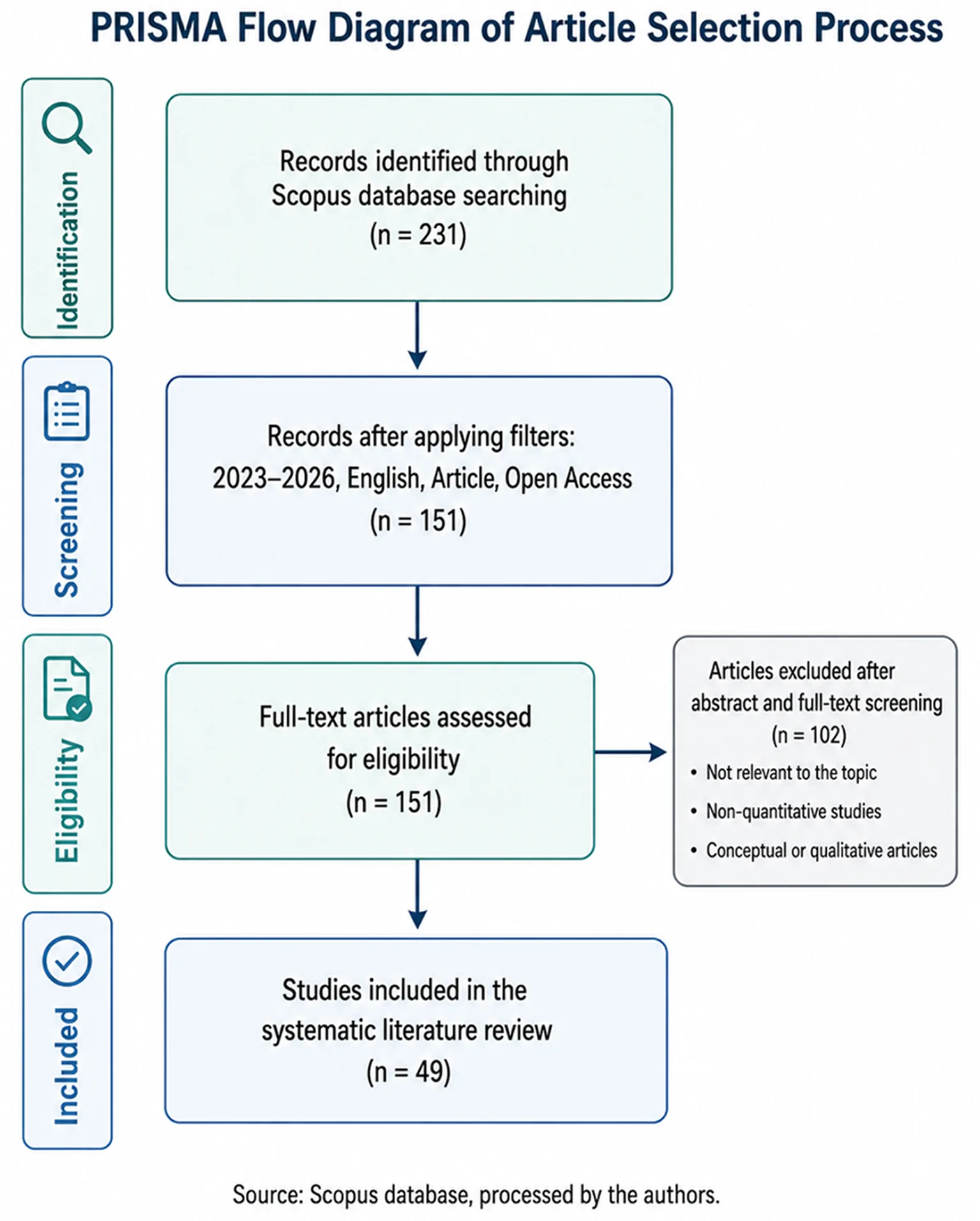
PRISMA Flow Diagram of Article Selection Process. Source: Scopus database, processed by the authors.

### 2.6 Data analysis and synthesis

The selected articles were analyzed using a descriptive and thematic synthesis approach. Descriptive analysis was used to present the distribution of studies based on publication year, research context, industry sector, methodological design, and analytical technique. Meanwhile, thematic synthesis was employed to identify recurring themes related to digital innovation and corporate sustainability. The synthesis focused on how digital innovation contributes to corporate sustainability, what mechanisms explain this relationship, and which contextual factors strengthen or weaken the effect.

The analysis was organized around several key aspects: (1) the role of digital innovation in improving sustainability performance; (2) the relationship between digitalization and environmental, social, and economic sustainability; (3) the mediating and moderating variables used in previous studies; (4) the dominant theoretical foundations applied in the literature; and (5) research gaps that provide opportunities for future studies. Through this process, the review not only summarizes existing empirical evidence but also provides a structured understanding of how digital innovation contributes to corporate sustainability in recent literature.

## 3. Results

The final review included 49 quantitative empirical articles examining the relationship between digital innovation and corporate sustainability. The selected studies show that this research stream has developed around several dominant themes, including digital transformation, digital finance, artificial intelligence, supply chain digitalization, digital–green integration, and digital governance. Most studies reported a positive relationship between digital innovation and sustainability outcomes, although several articles also showed that this relationship is nonlinear, conditional, or dependent on organizational and institutional readiness. The complete synthesis of 49 articles can be seen in Table 2.

**Table 2.** The complete synthesis of 49 articles.

No	Author(s)	Research Focus/Objective	Context and Sample	Method and Analysis	Main Findings	Research Gap/Remaining Gap
1	Al-Taani et al. (2024)	Investigates the contribution of digital accounting practices and digitally administered zakat to organizational sustainability, particularly through improved accountability, transparency, and philanthropic resource management.	Firms using Islamic and digital accounting practices in Malaysia and the Middle East; 328 experienced participants.	Quantitative survey using structured questionnaires; data analyzed with SmartPLS 4.	Both digital accounting and digital zakat were associated with higher corporate sustainability. Digital accounting demonstrated the stronger relationship, whereas digital zakat showed a smaller but statistically significant contribution. These findings suggest that digitalized financial practices may support sustainability through more transparent and accountable resource management.	The study does not fully explain how digital accounting and digital zakat interact in producing sustainability outcomes. Evidence also remains limited to particular geographical and organizational settings. Further research should test the underlying mechanisms across additional industries and institutional contexts and examine whether financial transparency strengthens or conditions these relationships.
2	Alshehadeh et al. (2024)	Examines how the adoption of digital accounting and the integration of digital zakat contribute to corporate sustainability, while considering financial transparency as a factor that may strengthen these relationships.	Malaysian specialists; 298 respondents.	Quantitative survey; SEM-PLS using SmartPLS 4.0.	Digital accounting, digital zakat, and financial transparency contribute positively to corporate sustainability. Financial transparency emerges as the most influential factor and also strengthens the sustainability benefits associated with digital accounting and digital zakat. This indicates that technological adoption generates stronger sustainability outcomes when it is accompanied by transparent financial practices.	The study integrates several previously separated concepts into a single sustainability model. Nevertheless, its evidence remains concentrated on Islamic financial practices and Malaysian respondents. Further research should examine wider forms of digital innovation across different industries, institutional environments, and national settings.
3	Alsaffarini & Awwad (2026)	Investigates the combined contribution of green marketing and artificial intelligence to the environmental, social, and economic dimensions of corporate sustainability.	Palestinian industrial sector; 500 valid survey respondents and 15 interview participants.	Explanatory sequential mixed-methods; quantitative phase analyzed using PLS-SEM, supported by semi-structured interviews and thematic analysis.	Green marketing and artificial intelligence improve all three dimensions of corporate sustainability. Artificial intelligence also strengthens the contribution of green marketing to sustainability, although its moderating influence remains limited. The findings suggest that AI can support green marketing initiatives, but its sustainability value depends on how effectively it is integrated into organizational practices.	The study extends evidence from a developing and resource-constrained setting, but the findings remain specific to Palestinian industrial firms. Further studies are needed to determine whether similar relationships occur across countries, sectors, and different levels of artificial intelligence capability.
4	Dhyanasaridewi, Murwaningsari, & Mayangsari (2024)	Examines whether sustainability innovation, proactive sustainability strategies, and digital transformation improve corporate sustainability performance and whether organizational culture changes the strength of these relationships.	Non-financial companies listed on the Indonesia Stock Exchange (IDX) during 2018–2022; 705 firm-year observations from financial statements, annual reports, and sustainability reports.	Quantitative study using secondary data; content analysis and multiple regression analysis; size and leverage used as control variables.	Sustainability innovation, proactive sustainability strategies, and digital transformation are associated with stronger corporate sustainability performance. However, organizational culture does not strengthen these relationships. This suggests that sustainability-oriented practices may directly improve performance, while the role of culture may depend on more specific organizational conditions.	Previous evidence concerning sustainability innovation and performance remains inconsistent, while earlier research has often examined only selected dimensions of sustainability strategy. The unsupported role of organizational culture also indicates a need for more precise cultural measures and deeper analysis of the conditions under which culture supports sustainability transformation.
5	Fan, Zhang, Zhang, Kang, & Cui (2025)	Investigates whether the integration of digital technologies with real economic activities improves corporate ESG performance and whether organizational inertia limits this contribution.	Chinese A-share listed companies from 2009 to 2023; 11,034 firm-year observations.	Quantitative panel-data study; fixed-effects regression, robustness checks, moderation testing, and heterogeneity analysis across ownership, region, and industry type.	Digital–real technology integration improves overall ESG performance, while organizational inertia weakens its positive contribution. The benefits are more evident among non-state-owned companies, firms located in central and western China, and companies operating outside highly polluting industries. The improvement is concentrated in environmental and governance performance, with no clear contribution to the social dimension.	Existing research commonly treats digitalization as a general organizational process and gives less attention to its integration with real operational activities. Further research should clarify the mechanisms linking this integration to ESG outcomes and examine internal constraints beyond organizational inertia. More comprehensive measures are also needed to capture process, structural, and managerial forms of integration that cannot be represented through patent data alone.
6	Fei, Liou, & Sun (2025)	Explores how artificial intelligence and robotic process automation affect environmental performance, operational efficiency, and brand equity among service-sector SMEs.	Three award-winning Taiwanese service SMEs participating in a national AI transformation program; supported by ERP records, carbon audits, and a structured survey of 121 employees.	Mixed-methods case study; analysis based on ERP records, GHG Protocol-based carbon audits, survey data, and between-firm statistical comparisons.	Artificial intelligence and robotic process automation reduce resource consumption, operational waste, electricity use, and printing expenditure. Their adoption also improves digital marketing, customer engagement, brand recognition, customer loyalty, and perceptions of organizational innovation. These results show that automation technologies can simultaneously support environmental efficiency and market-related organizational value.	Evidence on the environmental and branding consequences of artificial intelligence and robotic process automation remains limited, particularly among service SMEs. Future research should involve larger samples and more diverse service industries while examining implementation challenges such as investment costs, employee adjustment, data governance, digital readiness, and workforce transition.
7	Guo, Chen, & Chen (2025)	Examines how digital transformation influences the sustainable development performance of listed energy companies and identifies the organizational mechanisms through which this influence occurs.	Chinese A-share listed energy companies from 2011–2023; 1,465 valid firm-year observations.	Quantitative panel-data study; two-way fixed effects model, mediation analysis, and heterogeneity analysis. Digital transformation was measured through Python-based text mining of annual reports.	Digital transformation improves the sustainable development performance of energy companies by easing financing constraints and increasing the efficiency of resource allocation. Its contribution is more substantial among smaller companies and firms operating in downstream energy activities.	Previous research provides conflicting conclusions about the sustainability consequences of digital transformation because digital technologies may improve efficiency while also increasing costs, energy use, and carbon emissions. Further research should assess whether the identified benefits remain consistent across industries, countries, ownership structures, and different levels of digital maturity.
8	Guo & Kuang (2025)	Investigates how supply-chain digitalization supports the sustainable development of new-energy companies and examines the organizational and market conditions that shape this relationship.	Chinese A-share listed new energy companies from 2010–2023; 799 firm-year observations.	Quantitative panel regression using annual report text mining; moderation analysis and mediation analysis.	Supply-chain digitalization improves corporate sustainability by strengthening green innovation and reducing dependence on a concentrated group of suppliers. Its contribution becomes stronger when companies receive greater analyst attention and provide equity incentives to executives. These findings indicate that digitalization supports sustainability not only through technology adoption but also through changes in supply-chain relationships and governance incentives.	Previous studies have generally assumed a direct relationship between digitalization and sustainability without adequately explaining how digital supply chains reshape organizational networks. Further research should examine the hidden costs of digital transformation, including technological limitations, platform dependence, data fragmentation, interoperability problems, and differences in managerial capability.
9	Han, Liu, Xu, & Liu (2025)	Examines whether reform of China’s corporate registration system improves corporate sustainability through changes in ESG performance. Within this review, the study provides evidence on the institutional conditions surrounding sustainability in a digitally developing economy rather than on digital innovation itself.	Chinese A-share IPO firms from 2016–2022; 5,942 observations.	Quasi-natural experiment using staggered difference-in-differences (DID), robustness tests, IV analysis, PSM-DID, mechanism analysis, and heterogeneity analysis.	The registration-system reform improves corporate ESG performance, particularly corporate governance. This improvement is associated with stronger market competition, more rational investor behavior, and enhanced sponsor reputation. The contribution is more visible among non-state-owned companies, less profitable firms, and companies operating in regions with higher levels of market development.	Research on registration-system reform has predominantly examined financial and capital-market consequences, leaving its sustainability implications less developed. However, because the study does not directly examine digital innovation, it should be treated as supporting evidence concerning the institutional environment rather than as core evidence of a digital innovation–sustainability relationship.
10	Hu, Zhu, Zhao, & Xu (2023)	Investigates the nonlinear relationship between digital finance and corporate sustainability performance and examines the role of organizational resilience in explaining this relationship.	Chinese listed companies from Shanghai and Shenzhen Stock Exchanges, 2011–2021; 23,843 firm-year observations from 2,592 enterprises.	Quantitative panel-data study; two-way fixed effects model, mediating effect model, robustness tests, and endogeneity checks.	Digital finance has a nonlinear relationship with corporate sustainability. At an early stage of development, it may constrain sustainability performance, but its contribution becomes positive after digital financial capacity reaches a sufficient level. This improvement occurs partly because digital finance reduces financial volatility and supports longer-term organizational performance. The positive effects are more apparent among smaller, non-state-owned companies and firms located in less economically developed cities.	Previous research has generally examined digital finance in relation to carbon emissions, green innovation, or macroeconomic outcomes rather than comprehensive corporate sustainability. Further research should explore differences across stages of digital-finance development, regional inequalities, and the organizational conditions that determine when digital finance begins to generate positive sustainability outcomes.
11	Ibrahim, Moubarak, & Badawy (2026)	Investigates how organizational artificial intelligence capability contributes to sustainable performance through digital green innovation and examines whether green finance access and managerial environmental awareness influence this process.	321 organizations; respondents were senior professionals in strategic roles, including C-level executives, vice presidents, and directors.	Multi-method approach combining PLS-SEM, machine learning, and explainable AI. Predictive models include random forest, support vector regression, multilayer perceptron, and one-dimensional convolutional neural networks; XAI techniques include SHAP and LIME.	Artificial intelligence capability does not directly improve sustainable performance unless it is translated into digital green innovation. Digital green innovation therefore serves as the central mechanism linking AI resources with sustainability outcomes, while managerial environmental awareness also emerges as an important predictor.	Earlier studies commonly examined the direct organizational effects of artificial intelligence without integrating green innovation, financing conditions, and managerial awareness. Further research should explore nonlinear relationships, sector-specific differences, and the conditions under which green finance and leadership awareness become meaningful drivers of sustainability.
12	Işık, Ongan, Islam, Yan, Alvarado, & Ahmad (2025)	Examines how economic growth, energy prices, climate-policy uncertainty, and digitalization shape ESG performance across different levels of sustainability achievement in the United States.	United States macro-level context; the study analyzes ESG performance across different quantiles rather than focusing only on average effects.	Quantile and Quantile-on-Quantile analysis to examine whether the effects of economic, technological, and policy variables differ across low, medium, and high ESG performance levels.	Economic growth and energy prices are associated with stronger ESG performance, whereas climate-policy uncertainty has no consistent overall influence. Digitalization is associated with weaker ESG performance, suggesting that rapid technological development may lead firms to prioritize profitability and operational efficiency over ESG considerations.	Previous studies generally examined economic, technological, and policy variables separately. Further research should address ESG measurement inconsistencies, potential reciprocal relationships, and the limitations of using a single indicator to represent digitalization.
13	Ji, Zhou, & Zhang (2023)	Investigates whether digital transformation strengthens corporate sustainability and examines operational efficiency and organizational innovation as the mechanisms through which this effect occurs.	Chinese A-share listed companies during 2014–2020; 11,294 firm-year observations.	Quantitative panel-data study; fixed-effects estimation, 2SLS, mediation analysis, heterogeneity analysis, and Shapley value decomposition.	Digital transformation improves corporate sustainability by strengthening organizational innovation and operational efficiency. The contribution is more pronounced among private companies, manufacturing firms, and organizations located in eastern China.	Previous research on corporate sustainability was dominated by internal governance and managerial perspectives, with less attention to digital transformation as a strategic driver. Further studies should include unlisted firms, more recent periods, developed economies, and different institutional contexts.
14	Li & Zhao (2024)	Examines how digital transformation enables corporate sustainability by improving internal organizational efficiency and the external allocation of financial resources.	Chinese A-share listed companies from 2009–2022.	Quantitative panel fixed-effects model; mechanism testing, heterogeneity analysis, robustness testing, endogeneity checks, and supply-chain spillover analysis.	Digital transformation improves corporate ESG performance by increasing internal productivity and facilitating more efficient financial resource allocation. It also generates positive sustainability spillovers for suppliers and customers connected to the focal firm.	Existing studies have generally examined green innovation, disclosure quality, or external market attention as separate mechanisms. Further research should test the combined internal and external efficiency mechanisms across different industries, countries, and institutional environments.
15	Li & Jin (2024)	Investigates how the intensity of artificial intelligence adoption affects corporate sustainability performance and examines organizational change and digital capability as complementary organizational conditions.	Manufacturing enterprises in China; 451 employee survey responses from six manufacturing firms in eastern, southern, and central China.	Quantitative survey; confirmatory factor analysis, reliability and validity testing, regression analysis, mediation testing, moderation testing, and moderated mediation analysis using SPSS PROCESS Macro.	More intensive artificial intelligence adoption improves corporate sustainability performance by stimulating organizational change. Stronger digital capability enables firms to translate AI adoption into organizational transformation more effectively.	Earlier studies commonly examined artificial intelligence and sustainability separately and gave limited attention to organizational change as an explanatory mechanism. The model should be validated across broader industries, countries, and alternative sources of organizational data.
16	Li, Li, & Wu (2025b)	Examines how digital trade contributes to corporate ESG performance through technological innovation, international expansion, and improved information transparency.	Chinese listed firms from 2010–2022.	Quantitative panel-data study; mediation analysis, instrumental variable approach, propensity score matching, Heckman selection method, and heterogeneity analysis.	Digital trade improves overall ESG performance, particularly environmental and social outcomes. Technological innovation represents the primary mechanism, while internationalization and information transparency provide additional pathways.	Existing research has mainly examined general digital transformation rather than the specific sustainability implications of digital trade. Further research should assess whether these ESG benefits apply across different countries, institutional systems, ownership structures, and stages of organizational development.
17	Li, Ma, & Jin (2025a)	Investigates whether more efficient supply-chain operations improve corporate sustainability and whether digitalization and internal governance strengthen this contribution.	Chinese A-share listed companies from 2010–2022; 8,000 firm-year observations.	Quantitative panel-data study; two-way fixed effects model using Stata and Python-based text analysis.	Greater supply-chain efficiency improves sustainable corporate growth by reducing inventory duration and logistics-related costs. Digitalization and stronger internal governance increase the sustainability benefits of efficient supply-chain operations.	Earlier research concentrated primarily on the financial consequences of supply-chain management and commonly relied on broad performance indicators. Further studies should examine external influences such as government regulation, market disruption, and industry-level change.
18	Liu, Song, & Fu (2025a)	Examines how digital finance improves corporate ESG performance through the mutually reinforcing roles of digital transformation and green technological innovation.	Chinese A-share listed firms from 2011–2023.	Quasi-natural experiment using China’s FinTech Innovation Regulatory Pilot Policy; staggered DID model, robustness checks, Bartik instrumental variable, mechanism analysis, moderation analysis, and heterogeneity analysis.	Digital finance strengthens corporate ESG performance through digital transformation and green technological innovation. These mechanisms reinforce one another and generate stronger sustainability benefits when developed together.	Previous studies commonly examined financing constraints, digital transformation, or green innovation as separate mechanisms. Further research should clarify how digital and green capabilities interact across institutional settings, organizational life-cycle stages, regions, and environmental risk profiles.
19	Liu, Song, Zhou, & Liu (2025b)	Investigates how artificial intelligence adoption affects corporate ESG performance through green innovation and supply-chain efficiency and examines digital transformation as an enabling organizational condition.	Chinese listed firms from 2011–2023; 22,953 firm-year observations.	Quantitative panel-data study; mechanism analysis, moderation analysis, and heterogeneity analysis.	Artificial intelligence adoption improves environmental, social, and governance performance by encouraging green innovation and increasing supply-chain efficiency. Companies with more advanced digital transformation obtain greater ESG benefits from AI adoption.	The organizational mechanisms through which artificial intelligence improves ESG performance remain insufficiently understood. Further studies should jointly examine green innovation, supply-chain efficiency, and digital maturity across broader industries, countries, and organizational conditions.
20	Lulaj & Brajković (2025)	Investigates how finance, accounting, and digital disruption influence the relationship between auditing, corporate sustainability, and ESG integration in financial reporting from a triple-helix perspective.	Kosovo; 200 experts from corporate, academic, and governmental sectors during 2024–Q1 2025.	Quantitative survey; exploratory factor analysis, confirmatory factor analysis, and moderation analysis using SPSS and AMOS.	Financial, accounting, and digital developments support stronger ESG integration within corporate and public-sector settings. However, weak interaction among governmental, academic, and corporate actors may limit the sustainability benefits of digital and financial innovation.	Previous research generally examined ESG reporting, auditing, sustainability, accounting, and digital disruption as separate areas. Further research should investigate how these elements interact across institutional sectors, particularly in developing and transition economies.
21	Lyu, Yang, & Zhang (2026)	Examines whether China’s Integration of Informatization and Industrialization policy strengthens corporate ESG performance and identifies the mechanisms through which the policy produces sustainability outcomes.	Chinese A-share listed companies; the study uses the IoII pilot policy as a quasi-natural experiment.	Difference-in-differences (DID), mechanism analysis, and heterogeneity analysis.	The policy improves corporate ESG performance, particularly in the environmental and governance dimensions. These benefits are generated through stronger green innovation, improved corporate governance, and greater information transparency, with more pronounced effects among high-technology firms, non-state-owned enterprises, and companies with lower growth rates.	Existing research has rarely examined the sustainability implications of policies integrating digital technologies with industrial development. Further studies should assess whether similar policy-driven digital integration produces comparable ESG benefits across countries, industries, and institutional settings.
22	Manogna, Singh, & Singh (2026)	Investigates whether the organizational adoption of green fintech improves ESG performance among publicly listed companies in India.	96 major Indian listed firms from 2014–2023.	Panel regression models, including pooled OLS, fixed effects, random effects, IV-2SLS, and system GMM.	Greater adoption of green fintech is associated with stronger ESG performance, indicating that sustainability-oriented financial technologies can support responsible corporate practices in emerging markets.	Previous studies have provided limited firm-level evidence on the relationship between green fintech and ESG performance. The proposed mechanisms involving financing efficiency, information transparency, and process optimization have not been tested directly and therefore require further empirical examination.
23	Neiroukh & Çağlar (2025)	Examines how the quality of management accounting information systems contributes to corporate sustainability through accurate financial reporting and evaluates the enabling roles of artificial intelligence and green corporate governance.	Jordanian commercial banks; 257 accounting and finance professionals.	Quantitative survey; mediation and moderation analysis grounded in Resource-Based View and Contingency Theory.	Higher-quality accounting information systems improve corporate sustainability both directly and through more accurate financial reporting. Artificial intelligence strengthens reporting accuracy, while green corporate governance enables organizations to translate information-system quality into stronger sustainability outcomes.	Research has paid limited attention to the indirect sustainability contribution of management accounting information systems. Further studies should examine the interaction among information-system quality, artificial intelligence, green governance, and sustainability across industries, countries, and different levels of digital maturity.
24	Nguyen, Do, & Phung (2026)	Examines how green and social investment contributes to corporate sustainability through sustainable organizational behavior and evaluates digitalization as a supporting condition.	Maritime and logistics firms in Vietnam; 225 participants from 65 firms.	Quantitative survey; single-level PLS-SEM, with assessment of nested data structure through intraclass correlation coefficients.	Green and social investment encourages pro-environmental and pro-social organizational behavior, which subsequently improves sustainability reporting and business performance. Digitalization strengthens these relationships by improving monitoring, transparency, operational efficiency, data accuracy, and stakeholder engagement.	Previous research commonly considered green and social investment separately and rarely positioned sustainable organizational behavior as an explanatory mechanism. Further studies should test the model across sectors, countries, and longitudinal settings to determine whether digitalization consistently strengthens sustainability outcomes.
25	Novicka (2025)	Investigates how big-data analytics capability supports sustainable business performance through readiness for digital sustainability reporting and the integration of digitalization with sustainability.	Large Latvian organizations preparing for CSRD-based digital sustainability reporting; 75 responses.	Quantitative survey using PLS-SEM; supported by content analysis of open-ended responses.	Big-data analytics capability supports business performance by enabling organizations to develop digital sustainability-reporting readiness and integrate digital capabilities with sustainability practices. Sustainability and digitalization do not automatically create performance benefits unless organizations possess the reporting capabilities needed to convert them into business value.	Existing literature does not sufficiently explain how digital sustainability-reporting capability transforms sustainability initiatives into organizational performance. Further validation is needed across larger samples, other European countries, and organizations with more mature digital reporting systems.
26	Oh, Cho, & Park (2025)	Compares the contribution of conventional innovation and digital innovation to the sustainability of venture firms.	South Korean venture firms; 3,000 observations from secondary survey data collected by the Korean Ministry of SMEs and Startups and the Korea Venture Business Association in 2022.	Quantitative study using OLS regression; reliability testing, correlation analysis, VIF test, and multiple regression analysis.	Both conventional and digital innovation support venture-firm sustainability, but innovation in digital business models provides the strongest contribution. Conventional technological capability also remains important, whereas general organizational capability and product alignment with Industry 4.0 do not independently improve sustainability.	Earlier studies usually assessed conventional and digital innovation separately rather than comparing their relative effects. Further research should develop more comprehensive measures of strategic, operational, and Industry 4.0 capabilities in venture firms.
27	Pu (2025)	Examines how patent-based digital transformation affects corporate ESG performance and whether financial constraints limit its sustainability contribution.	Chinese A-share listed firms from 2012–2021; 1,157 firms and 9,479 firm-year observations.	Quantitative panel-data study using firm and year fixed effects, moderation analysis, industry heterogeneity analysis, ESG sub-dimension analysis, and robustness checks.	Digital transformation improves overall ESG performance, particularly environmental and social outcomes, but does not produce a clear improvement in corporate governance. Financial constraints reduce firms’ ability to translate digital transformation into ESG benefits, while traditional industries obtain greater marginal gains from digital upgrading.	Previous research often relied on aggregate ESG measures and text-based proxies for digital transformation, potentially obscuring dimension-specific outcomes. Further studies should explain why governance performance is less responsive and identify how financially constrained firms can obtain greater sustainability benefits from digital innovation.
28	Samidi & Kwan (2024)	Investigates how company characteristics, board practices, digital transformation, Shariah compliance, and gender diversity influence ESG ratings among Malaysian listed firms.	Bursa Malaysia-listed companies from 2018–2022; final sample of 40 firms after screening for complete ESG rating data.	Panel regression analysis using secondary data from Bloomberg and company annual reports.	Board gender diversity improves ESG ratings, while larger organizational size is associated with weaker ESG performance. Board diligence and digital transformation do not independently improve ESG ratings, suggesting that technological investment alone may be insufficient without complementary governance and reporting capabilities.	Evidence concerning the determinants of ESG ratings remains inconsistent, particularly in emerging markets. Further research should examine whether digital transformation produces indirect or conditional effects through governance quality, disclosure practices, stakeholder pressure, and sectoral readiness.
29	Sang, Loganathan, & Lin (2026)	Examines whether China’s National Artificial Intelligence Innovation and Development Pilot Zone policy improves corporate sustainable development and identifies the organizational capabilities supporting this effect.	Chinese listed companies from 2014–2024; AI Pilot Zone policy used as a quasi-natural experiment.	Multi-period difference-in-differences approach, supported by parallel trend tests, placebo tests, PSM-DID, robustness checks, mechanism analysis, and heterogeneity analysis.	The AI policy improves corporate sustainable development by strengthening firms’ innovation, adaptation, and knowledge-absorption capabilities. Its benefits are greater among organizations with stronger research intensity, more effective internal controls, and better access to digital financial infrastructure.	Previous AI research has focused mainly on productivity, efficiency, and financial performance rather than the sustainability consequences of public AI policy. Further studies should test comparable policies in other countries and assess whether their sustainability benefits persist over longer periods.
30	Sari, Arief, Hamsal, & Rahim (2024)	Examines how intellectual capital, digital innovation, and strategic alliances contribute to corporate sustainability performance in Indonesia’s information and communication technology industry.	ICT companies in Indonesia; data collected from more than 90 ICT companies.	Quantitative survey using purposive sampling; data analyzed with SmartPLS 4.0.	Digital innovation directly improves corporate sustainability and also supports the formation of strategic alliances. Intellectual capital contributes indirectly by strengthening alliance development, while strategic alliances provide an additional pathway for improving sustainability performance.	Integrated research on intellectual capital, digital innovation, strategic alliances, and sustainability remains limited in Indonesia’s ICT sector. Further studies should use broader samples and longitudinal designs and examine the mediating role of strategic alliances across different digital industries and institutional contexts.
31	Sharma & Radhika (2026)	Examines whether digital transformation serves as a structural driver of ESG performance among publicly listed companies in India.	Indian BSE 500 firms from April 2022 to March 2024; final sample of 301 firms and 602 firm-year observations.	Panel regression analysis using a fixed-effects model; digital transformation measured through text mining and word frequency analysis of annual reports.	Digital transformation improves overall ESG performance, with the strongest contribution appearing in the governance dimension. The findings indicate that digitalization supports sustainability mainly by reducing information asymmetry, increasing transparency, and strengthening regulatory compliance.	Empirical evidence on the digital transformation–ESG relationship in India remains limited. Further studies should employ longer observation periods, compare sectors, and examine the specific mechanisms through which digital transformation affects each ESG dimension.
32	Su & Liang (2025)	Examines how digital finance contributes to corporate ESG performance through productivity improvement and external analyst monitoring.	Chinese listed firms from 2011–2023; 22,576 firm-year observations.	Fixed-effects mediation model using corporate data, the Peking University Digital Financial Inclusion Index, and Huazheng ESG scores.	Digital finance improves ESG performance directly and indirectly by increasing total factor productivity and attracting greater analyst attention. These findings show that digital finance supports sustainability through both internal efficiency and stronger external oversight.	Previous studies more commonly examined how ESG performance affects productivity or analyst coverage rather than the reverse pathway. Further research should assess individual ESG dimensions and examine boundary conditions such as ownership, institutional quality, regional development, and market maturity.
33	Sutisna, Hidayat, Affandi, Komaruddin, & Pratama (2025)	Examines how digital technology literacy and digital economy literacy influence the sustainability of micro and small businesses through the Technology Acceptance Model.	Micro and small business owners in Banten Province, Indonesia; 162 respondents.	Quantitative survey; PLS-SEM based on the Technology Acceptance Model.	Digital technology literacy strengthens business sustainability, while digital economy literacy does not produce a clear direct effect. Perceived usefulness and ease of use encourage the adoption of digital tools, which subsequently supports more sustainable business operations.	The evidence is limited to micro and small businesses in Banten and is dominated by culinary enterprises. Future studies should use broader sectoral samples and more comprehensive measures of digital economy literacy, including digital marketing and platform-based business capabilities.
34	Tan & Cui (2026)	Investigates whether digital innovation improves corporate sustainability and whether economic policy uncertainty changes the strength of this relationship.	Chinese A-share listed firms from 2014–2023.	Quantitative panel-data study; robustness tests, DID-based validation, mechanism testing, heterogeneity analysis, and patent-based digital innovation analysis.	Digital innovation enhances corporate sustainability by improving managerial efficiency and reducing short-term managerial orientation. Economic policy uncertainty strengthens this contribution, while firms with stronger green transformation capability and those operating in more developed markets obtain greater sustainability benefits.	Existing research often examines the economic or environmental effects of digital innovation separately rather than treating corporate sustainability as an integrated outcome. Further studies should develop more accurate measures of emerging technologies, including generative artificial intelligence, blockchain, and other disruptive digital innovations.
35	Tian, Song, Qu, & Li (2025)	Examines how ESG performance contributes to corporate value through innovation capability and whether digitalization strengthens this relationship in the textile and apparel industry.	Chinese listed textile and apparel companies from 2013–2023; 408 firm-year observations.	Quantitative panel-data study using two-way fixed effects, mediation testing, and moderation testing.	Stronger ESG performance increases corporate value, partly by enhancing organizational innovation. Digitalization strengthens the relationship between ESG performance and corporate value but does not significantly improve the innovation pathway.	The study does not directly test digital innovation as an antecedent of corporate sustainability because digitalization is positioned as a moderating factor. Further research should examine whether more advanced digital technologies and stronger internal digital capabilities enable ESG practices to generate innovation more effectively.
36	Wang, Li, & He (2024)	Examines whether digital finance produces local and cross-regional spillover effects on corporate ESG performance.	Chinese city–enterprise matched data from 2011–2022.	Spatial econometric analysis to test local and neighbouring regional effects; mechanism and heterogeneity analysis.	Digital finance improves corporate ESG performance within the focal region and in neighbouring areas. These spillovers occur through the movement of labour and capital and the diffusion of technology, with stronger effects in regions possessing better digital infrastructure and larger economic capacity.	Previous studies primarily examined the direct local effects of digital finance and gave limited attention to cross-regional sustainability spillovers. Further research should investigate how institutional quality, infrastructure, and regional development differences shape these spatial effects.
37	Wang, Zhang, & Zhang (2026)	Examines how the ideological background of chief executive officers affects corporate sustainability through green innovation, digital innovation, and integrated green–digital innovation.	Chinese listed firms from 2012–2021; 7,645 observations from 1,633 firms.	Quantitative panel-data analysis grounded in upper echelons theory and managerial discretion literature; mediation and moderation analysis.	CEOs with stronger political affiliation improve environmental and social sustainability partly by encouraging green, digital, and integrated green–digital innovation. Greater managerial discretion strengthens the ability of innovation activities to generate sustainability outcomes.	Digital innovation is not the primary independent variable because the study focuses on CEO characteristics. Further research should determine whether the identified innovation pathways remain applicable outside China and in organizational settings without politically embedded leadership structures.
38	Wang & Zhang (2025)	Examines how financial technology contributes to corporate sustainability through supply-chain finance and evaluates the influence of organizational and environmental conditions.	China’s A-share non-financial listed companies from 2012–2022.	Panel-data analysis using fixed-effects, mediation-effect, moderation-effect, and heterogeneity models.	Financial technology improves corporate sustainability directly and indirectly by strengthening supply-chain finance. The benefits are greater among firms with mature digital capabilities, organizations in technology-intensive industries, and companies operating in regions with stronger digital infrastructure and market institutions.	Existing studies often emphasize direct technology effects and overlook organizational mechanisms and environmental boundary conditions. Further research should examine how regulatory systems, supply-chain structures, and regional digital readiness shape the sustainability benefits of financial technology.
39	Xu & Xu (2025)	Examines how government open-data platforms influence corporate ESG performance.	Chinese A-share listed firms from 2007–2023; city-level staggered implementation of government open data platforms.	Staggered difference-in-differences approach; robustness tests, mechanism analysis, and heterogeneity analysis.	Government open-data platforms improve ESG performance by reducing perceived uncertainty and increasing external stakeholder attention. The benefits are stronger where platform quality is higher and vary according to ownership structure and industry environmental intensity.	Previous research has concentrated mainly on firm-level digital transformation and paid less attention to government digital governance. Further studies should compare different types and quality levels of public data platforms and examine their effects across industries, ownership structures, and institutional environments.
40	Yang & Luo (2024)	Examines how the integration of digital and green transformation strengthens corporate sustainable development through resource, technological, and governance mechanisms.	Chinese A-share listed companies from 2010–2022.	Quantitative panel-data analysis; coupling coordination model, endogeneity tests, robustness tests, mechanism analysis, and heterogeneity analysis.	Digital–green integration improves sustainable development by encouraging green technological innovation, increasing information transparency, and improving labour-resource allocation. Its contribution is stronger among larger firms, state-owned enterprises, and companies operating in industries with lower environmental uncertainty.	Previous studies commonly examined digital transformation and green transformation separately. Further research should test the combined digital–green mechanism across countries, industries, organizational sizes, and different levels of environmental uncertainty.
41	Yao, Zhang, Sun, & Bo (2025)	Examines how digital transformation contributes to corporate sustainability through internal managerial incentives and external analyst monitoring.	Chinese A-share listed companies from 2012–2022; 26,085 valid observations.	Quantitative panel regression with year and industry fixed effects; mediation analysis, robustness tests, and heterogeneity analysis.	Digital transformation improves corporate sustainability by encouraging management through compensation incentives and strengthening external oversight through analyst attention. The benefits are more apparent among firms located in eastern regions and companies outside the growth stage.	Previous research has largely emphasized direct effects, operational efficiency, and innovation mechanisms. Further studies should examine broader internal and external governance pathways, adopt more comprehensive sustainability measures, and test the relationships beyond Chinese listed firms.
42	Yilmaz, Aksoy, Tatoglu, & Bayraktar (2025)	Examines the nonlinear relationship between digital maturity and corporate sustainability efficiency in an emerging-market context.	Non-financial firms listed on Borsa Istanbul, Turkey; 30 firms from 2018–2022.	Text mining to construct a corporate digitalization index; data envelopment analysis to measure corporate sustainability efficiency; panel regression analysis.	Digital maturity initially reduces sustainability efficiency because firms face substantial investment, implementation, and adaptation costs. Once digital capabilities reach a more advanced stage, however, organizations begin to obtain operational and strategic benefits that improve sustainability efficiency.	Earlier studies often treated digital transformation as a single adoption event and assumed an immediate positive sustainability effect. Further research should examine capability-development stages, transition costs, and nonlinear effects across different emerging economies and organizational contexts.
43	Yin, Su, & Ding (2024)	Investigates the nonlinear influence of digital finance on corporate ESG performance and examines the mechanisms and contextual conditions shaping this relationship.	Chinese A-share listed companies from Shanghai and Shenzhen Stock Exchanges, 2011–2021.	Panel-data regression; nonlinear threshold analysis, mediation analysis, moderation analysis, heterogeneity analysis, and robustness tests.	Digital finance initially constrains ESG performance but becomes beneficial after firms or regions achieve sufficient digital-finance maturity. Green technological innovation and public environmental attention help explain this transition, while regulation, environmental uncertainty, and firm-level digital transformation shape the strength of the relationship.	Existing studies commonly assume a linear relationship between digital finance and ESG outcomes. Further research should identify maturity thresholds more precisely and examine how regulatory conditions, environmental uncertainty, and organizational digital readiness alter the sustainability effects of digital finance.
44	Ying & Jin (2023)	Examines how technology-oriented and market-oriented digital transformation affect corporate sustainability and whether ambidextrous innovation strengthens these relationships.	Chinese A-share listed companies from 2013–2021.	Two-way fixed-effects model and two-stage least squares method.	Both technological and market-based forms of digital transformation improve corporate sustainability. Exploratory and exploitative innovation further strengthen these effects, indicating that firms obtain greater sustainability value when digital transformation is supported by balanced innovation capabilities.	Previous research has often measured digital transformation as a single aggregate construct. Further studies should distinguish among its different dimensions and examine which innovation capabilities enable firms to convert digital initiatives into sustainable outcomes.
45	Zada, Zada, Dhar, Ping, & Sarkar (2025)	Examines how digital leadership promotes firm sustainability through green innovation and evaluates top-management innovativeness as an enabling condition.	Chinese information technology sector; 413 employees from diverse organizational roles.	Quantitative study with three-phase time-lagged data collection to reduce common method bias; hypothesis testing based on Organizational Learning Theory and Dynamic Capabilities Theory.	Digital leadership strengthens firm sustainability directly and indirectly by encouraging green innovation. The contribution of digital leadership to green innovation becomes stronger when senior managers are more receptive to experimentation, technological change, and new ideas.	Prior research has concentrated more heavily on organization-level digital transformation than on leadership-based mechanisms. Further studies should examine how digital leadership, managerial innovativeness, and green innovation interact across industries, countries, and organizational cultures.
46	Zhang, Wei, Feng, & Xu (2026b)	Examines how digitalization affects ESG performance among agricultural firms and evaluates the organizational and contextual conditions that shape this effect.	Chinese listed agricultural firms from 2013–2022.	Longitudinal panel-data study using Ordinary Least Squares regression; moderation and heterogeneity analyses.	Digitalization improves ESG performance by strengthening internal resource integration and information transparency. However, reliance on traditional organizational resources may reduce these benefits, suggesting that digital capabilities can substitute for less efficient conventional inputs. The effects also differ according to financial structure, market concentration, and regional conditions.	Research on digitalization and ESG performance remains concentrated in manufacturing, finance, and technology-intensive sectors. Further studies should examine how agricultural firms convert digital investment into sustainability under resource constraints, environmental vulnerability, and unequal regional digital infrastructure.
47	Zhang, Huang, Chen, & Chen (2025)	Investigates how policy-driven supply-chain digitalization affects corporate ESG performance and identifies the financial, technological, and human-capital mechanisms involved.	Chinese A-share listed firms from 2009–2023.	Difference-in-differences model using the Supply Chain Innovation and Application Pilot Policy as a quasi-natural experiment; mechanism and heterogeneity analyses.	Supply-chain digitalization improves ESG performance by easing financing constraints, stimulating digital innovation, and upgrading the composition of organizational human capital. Its contribution is stronger when firms possess supportive governance arrangements and receive greater market attention.	Previous research has provided limited causal evidence on the sustainability effects of supply-chain digitalization. Further studies should investigate the interaction between technological adoption, workforce restructuring, corporate governance, and supply-chain characteristics across different institutional environments.
48	Zhang, Wang, Zhu, & Song (2026a)	Examines how executives’ attention to digital issues influences corporate sustainability through organizational resource-orchestration capabilities.	Chinese A-share listed firms from 2012–2023.	Panel-data analysis with endogeneity and robustness tests; mechanism, moderation, heterogeneity, and dimension-focused analyses.	Executives’ digital attention improves sustainability by helping firms structure, combine, and deploy resources more effectively. Financial constraints weaken this contribution, whereas media attention strengthens it. The benefits are more evident in environmental performance than in short-term financial outcomes.	Existing studies have rarely examined managerial cognition as an antecedent of digital transformation and sustainability. Further research should clarify how executive attention is converted into resource-allocation decisions and long-term outcomes under different financing, media, and governance conditions.
49	Zhu & Li (2025)	Examines how the integration of digital and green transformation improves corporate sustainability and evaluates the role of environmental regulation.	Chinese publicly listed firms from 2015–2023.	Two-way fixed-effects model; mechanism analysis and moderation analysis.	Digital and green transformation jointly produce stronger sustainability benefits than either transformation would create independently. This synergy operates through more effective resource integration and innovation, while environmental regulation further strengthens the relationship. The benefits vary across pollution intensity, competitive conditions, and intellectual-property protection.	Previous studies have generally examined digital and green transformation separately. Further research should explain how organizations can operationalize their integration across different regulatory systems, market structures, environmental-risk levels, and intellectual-property regimes.

Overall, the results indicate that digital innovation contributes to corporate sustainability through multiple pathways rather than through a single direct effect. The strongest recurring mechanisms are green innovation, efficiency improvement, financial access, information transparency, supply chain coordination, and governance enhancement. However, the evidence also suggests that digital innovation does not automatically produce sustainability benefits. Its impact depends on digital maturity, organizational capability, leadership support, institutional environment, and the extent to which digital initiatives are integrated with sustainability-oriented strategies.

## 4. Discussion

### 4.1 Digital innovation as a multidimensional driver of corporate sustainability

The synthesis of the 49 selected articles shows that digital innovation has become a multidimensional driver of corporate sustainability. Rather than being limited to the adoption of digital technologies, digital innovation in the reviewed studies appears in diverse forms, including digital transformation, digital finance, FinTech, artificial intelligence, digital accounting, supply chain digitalization, digital trade, digital leadership, digital–green fusion, and government-led digital governance. This diversity indicates that the literature has moved beyond a narrow technological perspective and increasingly views digital innovation as a strategic, organizational, and institutional capability that enables firms to improve sustainability outcomes.

A substantial group of studies confirms that digital transformation enhances corporate sustainability by improving ESG performance, sustainable development performance, corporate value, and sustainability efficiency. Digital transformation strengthens firms’ ability to improve operational efficiency, allocate resources more effectively, increase innovation capability, and enhance information transparency ( Ji et al., 2023; Li & Zhao, 2024; Pu, 2025; Sharma & Radhika, 2026; Ying & Jin, 2023). Similar findings are also found in studies that examine digital transformation through executive incentives, analyst attention, ambidextrous innovation, and managerial mechanisms ( Yao et al., 2025; Tan & Cui, 2026; Zhang, Wang, Zhu, & Song, 2026a). These studies collectively indicate that digital transformation supports sustainability not only by upgrading technology, but also by reshaping managerial decision-making, governance quality, and organizational capability.

However, the findings also caution against assuming that digital innovation always generates immediate or linear sustainability benefits. Several studies reveal nonlinear, conditional, or insignificant relationships. For example, digital finance and digital maturity may follow a U-shaped relationship with ESG performance or sustainability efficiency, indicating that early-stage digitalization can produce adaptation costs before positive sustainability effects emerge ( Hu et al., 2023; Yin et al., 2024; Yilmaz et al., 2025). In the Malaysian context, digital transformation was not found to have a significant direct impact on ESG ratings, suggesting that digital adoption alone is insufficient without complementary governance and disclosure capabilities ( Samidi & Kwan, 2024). Similarly, macro-level evidence suggests that digitalization may negatively affect ESG performance when technological progress is not aligned with sustainability-oriented policy and corporate priorities ( Işık et al., 2025). Therefore, the reviewed evidence suggests that digital innovation is not automatically sustainable; it becomes sustainability-enhancing when firms possess adequate maturity, governance support, and strategic alignment.

The reviewed articles also show that digital innovation contributes to sustainability across different organizational and sectoral contexts. In the ICT sector, digital innovation improves sustainability directly and through corporate strategic alliances ( Sari et al., 2024). Among micro and small industries, digital technology literacy supports business sustainability by increasing technology acceptance and adoption intention ( Sutisna et al., 2025). In venture firms, digital business model innovation plays a stronger role than conventional innovation in supporting long-term sustainability ( Oh et al., 2025). Sector-specific studies also demonstrate that digitalization matters in agriculture, textile and apparel, maritime and logistics, banking, services, and energy firms ( Nguyen et al., 2026; Neiroukh & Çağlar, 2025; Tian et al., 2025; Zhang, Wei, Feng, & Xu, 2026b; Guo, Chen, & Chen, 2025). These findings imply that digital innovation is a cross-sectoral sustainability driver, but its form and mechanism differ according to industry characteristics, firm size, digital readiness, and institutional environment.

### 4.2. Mechanisms and boundary conditions in the digital innovation–sustainability relationship

The review reveals that the relationship between digital innovation and corporate sustainability is primarily explained through several recurring mechanisms. The first dominant mechanism is efficiency improvement. Digital transformation, digital finance, and FinTech improve sustainability by increasing operational efficiency, total factor productivity, resource allocation efficiency, and supply chain efficiency ( Ji et al., 2023; Li & Zhao, 2024; Su & Liang, 2025; Wang & Zhang, 2025). This efficiency pathway is important because sustainability requires firms to reduce waste, optimize resources, and balance economic and environmental goals. In this respect, digital innovation allows firms to pursue sustainability without necessarily sacrificing competitiveness.

The second mechanism is innovation capability, particularly green innovation, digital green innovation, and integrated green–digital innovation. Several studies demonstrate that digital technologies improve sustainability by enabling green technological innovation, digital technology innovation, and sustainability-oriented product or process innovation ( Ibrahim et al., 2026; Liu, Song, & Fu, 2025a; Liu, Song, Zhou, & Liu, 2025b; Yang & Luo, 2024; Zhu & Li, 2025). AI capability, for example, does not automatically enhance sustainability unless it is converted into digital green innovation ( Ibrahim et al., 2026). Similarly, AI adoption affects ESG performance through green innovation and supply chain efficiency, while digital transformation strengthens this relationship ( Liu, Song, Zhou, & Liu, 2025b). These findings suggest that digital innovation must be embedded in sustainability-oriented innovation processes to generate meaningful outcomes.

The third mechanism is transparency, accountability, and reporting quality. Studies on digital accounting, digital zakat, financial transparency, accounting information systems, and digital sustainability reporting show that digital innovation improves sustainability by enhancing financial transparency, accurate reporting, ESG disclosure, and stakeholder trust ( Al-Taani et al., 2024; Alshehadeh et al., 2024; Lulaj & Brajković, 2025; Neiroukh & Çağlar, 2025; Novicka, 2025). These findings are important because sustainability performance increasingly depends on credible reporting and verifiable information. Digital accounting and reporting systems enable firms to provide more transparent sustainability-related information, reduce information asymmetry, and strengthen accountability to stakeholders.

The fourth mechanism is financing access and market monitoring. Digital finance, FinTech, and green FinTech support ESG performance by reducing financing constraints, improving financial inclusion, increasing analyst coverage, and enhancing market monitoring ( Manogna et al., 2026; Su & Liang, 2025; Wang, Li, & He, 2024; Wang & Zhang, 2025). These findings show that financial digitalization helps firms overcome resource limitations that often hinder ESG investment. Digital finance also produces spatial spillover effects by enabling labor mobility, capital mobility, and technology diffusion across regions ( Wang, Li, & He, 2024). Thus, the sustainability effects of digital finance extend beyond individual firms and may create broader regional improvements in ESG practices.

The fifth mechanism concerns supply chain coordination and network-level digitalization. Supply chain digitalization and digital trade support sustainability through supply chain transparency, supplier coordination, digital technology innovation, human capital optimization, internationalization, and reduced supplier concentration ( Guo & Kuang, 2025; Li, Li, & Wu, 2025b; Li, Ma, & Jin, 2025a; Zhang, Huang, Chen, & Chen, 2025). These studies suggest that corporate sustainability is not only a firm-level outcome but also an inter-organizational outcome shaped by the digital integration of suppliers, customers, logistics providers, financial institutions, and regulators. Digitalized supply chains allow firms to monitor sustainability risks more effectively and coordinate sustainability practices across organizational boundaries.

The sixth mechanism is leadership, cognition, and governance. Digital leadership enhances firm sustainability through green innovation, while top management innovativeness strengthens this effect ( Zada et al., 2025). Executives’ digital attention improves sustainable development through resource structuring, resource bundling, and resource leveraging capabilities ( Zhang, Wang, Zhu, & Song, 2026a). CEO ideology and managerial discretion can also influence sustainability through green, digital, and integrated green–digital innovation pathways ( Wang, Zhang, & Zhang, 2026). These findings indicate that digital innovation is not purely technological; it depends on the ability of leaders and executives to interpret digital opportunities and direct resources toward sustainability goals.

The review also identifies important boundary conditions. Digital maturity, organizational inertia, financial transparency, top management innovativeness, digital capability, environmental regulation, economic policy uncertainty, media attention, marketization level, digital infrastructure, board diversity, CEO duality, and government policy all shape the strength of the digital innovation–sustainability relationship ( Fan et al., 2025; Li & Jin, 2024; Lyu et al., 2026; Sang et al., 2026; Xu & Xu, 2025; Yang & Luo, 2024). For example, organizational inertia can weaken the effect of digital–real technology integration on ESG performance ( Fan et al., 2025), while digital capability strengthens the impact of AI adoption on organizational change and sustainability performance ( Li & Jin, 2024). Government-led digital policies, such as AI pilot zones, open data platforms, and informatization–industrialization integration, improve ESG and sustainability outcomes by creating supportive institutional environments ( Lyu et al., 2026; Sang et al., 2026; Xu & Xu, 2025). These findings show that digital innovation produces stronger sustainability effects when supported by internal readiness and external institutional quality.

A particularly important insight from the review is the emergence of digital–green integration as a higher-order pathway to sustainability. Studies on digital–green fusion and digital and green transformation show that integrating digital and green strategies produces stronger effects than implementing them separately ( Yang & Luo, 2024; Zhu & Li, 2025). Digital–green fusion improves sustainability through green technology innovation, information transparency, and labor resource allocation efficiency, while digital and green transformation creates a multiplier effect through resource synergy and innovation synergy. These findings suggest that the future of corporate sustainability research should not only ask whether digital transformation improves sustainability, but also examine how digital transformation can be aligned with green transformation to create synergistic and long-term sustainability value.

### 4.3. Theoretical implications, practical implications, and future research agenda

The findings offer several theoretical implications. First, this review supports the Resource-Based View and Dynamic Capabilities Theory by showing that digital innovation functions as a strategic capability rather than merely a technological tool. Firms achieve sustainability when they can acquire, integrate, reconfigure, and deploy digital resources toward green innovation, operational efficiency, transparency, and long-term value creation ( Ibrahim et al., 2026; Li & Jin, 2024; Sang et al., 2026; Yang & Luo, 2024). Second, the findings extend stakeholder theory and agency theory by showing that digital innovation improves sustainability through transparency, disclosure quality, analyst attention, external monitoring, and accountability ( Al-Taani et al., 2024; Lulaj & Brajković, 2025; Su & Liang, 2025; Yao et al., 2025). Third, the review advances upper echelons theory, attention-based view, and resource orchestration theory by demonstrating that digital leadership, executive digital attention, CEO ideology, and top management innovativeness influence whether digital resources are translated into sustainability outcomes ( Wang, Zhang, & Zhang, 2026; Zada et al., 2025; Zhang, Wang, Zhu, & Song, 2026a).

The findings also support the Technology–Organization–Environment perspective. Digital innovation improves sustainability when technological capability is combined with organizational mechanisms and supportive environmental conditions ( Wang & Zhang, 2025). Similarly, government open data platforms, AI pilot zones, digital finance infrastructure, and supply chain innovation policies indicate that institutional digitalization can act as a soft governance mechanism that supports sustainability ( Sang et al., 2026; Xu & Xu, 2025; Zhang, Huang, Chen, & Chen, 2025). Therefore, corporate sustainability should be understood as a multilevel outcome shaped by the interaction between technology, organization, leadership, supply chains, and institutional systems.

For practice, the review suggests that firms should avoid treating digital innovation as a purely technical modernization project. Digital innovation should be embedded in sustainability strategy, green innovation, ESG reporting, supply chain coordination, and governance reform. Firms need to strengthen digital capabilities, leadership commitment, digital literacy, organizational change readiness, and green innovation capability to convert digital investment into sustainability performance. The evidence also suggests that firms should carefully manage the early-stage costs of digital transformation, since digital maturity and digital finance may produce nonlinear effects before sustainability benefits emerge ( Hu et al., 2023; Yin et al., 2024; Yilmaz et al., 2025).

For policymakers, the results indicate that digital infrastructure, digital finance, open data platforms, AI policy, and supply chain digitalization policies can support corporate sustainability when they reduce information frictions, improve financing access, and strengthen stakeholder oversight. However, policymakers should also recognize that digitalization may generate unequal effects across regions, sectors, and firm types. Firms in less developed digital ecosystems may require stronger institutional support, capacity-building programs, and financing mechanisms to benefit from digital innovation. Environmental regulation, intellectual property protection, marketization, and digital infrastructure should therefore be aligned with digital transformation policies to maximize sustainability outcomes.

For future research, several directions emerge. First, future studies should expand beyond China, as many reviewed articles focus on Chinese listed firms. Comparative cross-country studies are needed to assess whether the digital innovation–sustainability relationship differs across institutional environments, regulatory systems, and ESG maturity levels. Second, future research should develop more precise measures of digital innovation. Many studies still rely on broad indicators such as digital transformation indexes, annual report text mining, or digital finance indexes. Future studies should distinguish among AI capability, blockchain adoption, digital platform integration, digital maturity, green digital innovation, and digital governance quality.

Third, future research should examine nonlinear and time-lagged effects. The U-shaped findings in several studies suggest that the sustainability benefits of digital innovation may appear only after firms pass a maturity threshold. Longitudinal research should therefore examine transformation stages, adaptation costs, and the time required for digital investments to generate sustainability outcomes. Fourth, future studies should explore potential negative consequences of digital innovation, including higher energy consumption from digital infrastructure, digital inequality, data governance risks, cybersecurity problems, and digital greenwashing. Finally, future research should develop multilevel models integrating individual cognition, organizational capabilities, supply chain ecosystems, regional infrastructure, and institutional policy. This would provide a more complete understanding of how digital innovation contributes to sustainable corporate development.

## 5. Conclusion

This systematic literature review examined 49 quantitative empirical articles on the relationship between digital innovation and corporate sustainability. The findings indicate that digital innovation has become an important driver of sustainability outcomes, particularly through digital transformation, digital finance, artificial intelligence, supply chain digitalization, digital accounting, digital leadership, and digital–green integration. Overall, most studies show that digital innovation positively contributes to ESG performance, sustainable development performance, corporate sustainability performance, and sustainability efficiency.

The review also reveals that the effect of digital innovation on corporate sustainability is not automatic or universally linear. Its impact depends on several mechanisms, including green innovation, operational efficiency, financing access, information transparency, supply chain coordination, resource orchestration, and governance improvement. In addition, the relationship is shaped by contextual factors such as digital maturity, organizational capability, leadership support, environmental regulation, digital infrastructure, and institutional quality.

This study contributes to the literature by synthesizing recent empirical evidence and showing that digital innovation should be understood not merely as technological adoption, but as a strategic capability for achieving sustainable corporate development. Future research is encouraged to conduct more cross-country comparisons, develop more precise measures of digital innovation, examine nonlinear and time-lagged effects, and explore the potential negative consequences of digitalization, including digital inequality, energy consumption, and digital greenwashing.

## Ethical approval

Ethical approval was not required for this study because it is a systematic literature review based entirely on previously published and publicly accessible sources. The study did not involve human participants, animals, primary data collection, or the processing of identifiable personal information.

## Informed consent

Informed consent was not applicable, as this study did not involve human participants.

## Data Availability

Underlying Data Figshare: Dataset for “How Does Digital Innovation Drive Corporate Sustainability? A Systematic Literature Review” ( Prasetiyanto et al., 2026) DOI: 10.6084/m9.figshare.33047246 The project contains the following underlying data:
• List of Selected Articles for Analysis • PRISMA Checklist • PRISMA Flow Diagram Data are available under the terms of the Creative Commons Attribution 4.0 International license (CC-BY 4.0).
