# Supplementary material for: How Does Digital Innovation Drive Corporate Sustainability? A Systematic Literature Review
Source: F1000Res. 2026 Oct 26;15:1372. Originally published 2026 Aug 14. [Version 2] doi: 10.12688/f1000research.187511.2 (PMC13717168; doi:10.12688/f1000research.187511.2)
Supplement: Peer review history

# Reviewer report: Reviewer response for version 2

**DOI:** 10.5256/f1000research.212817.r533934

**Published:** 2026-10-28

**Authors:** Eka Desy Purnama

**Affiliations:** 1 Krida Wacana Christian University, Jakarta, Jakarta, Indonesia

The author has substantially revised the manuscript.

Are the rationale for, and objectives of, the Systematic Review clearly stated?

Yes

Is the statistical analysis and its interpretation appropriate?

Not applicable

If this is a Living Systematic Review, is the ‘living’ method appropriate and is the search schedule clearly defined and justified? (‘Living Systematic Review’ or a variation of this term should be included in the title.)

Not applicable

Are sufficient details of the methods and analysis provided to allow replication by others?

Partly

Are the conclusions drawn adequately supported by the results presented in the review?

Yes

Reviewer Expertise:

Business, Economic Management, and Financial Management

I confirm that I have read this submission and believe that I have an appropriate level of expertise to confirm that it is of an acceptable scientific standard.

# Reviewer report: Reviewer response for version 1

**DOI:** 10.5256/f1000research.206951.r528443

**Published:** 2026-10-13

**Authors:** Eka Desy Purnama

**Affiliations:** 1 Krida Wacana Christian University, Jakarta, Jakarta, Indonesia

This manuscript presents a relevant and timely systematic literature review on the relationship between digital innovation and corporate sustainability. The article is well-structured, the research question is clearly formulated, and the findings provide a useful synthesis of the mechanisms and contextual conditions that influence the relationship between digital innovation and sustainability outcomes. Overall, this manuscript has good scientific merit and is worthy of publication. However, there are several minor revisions that need to be made to improve methodological transparency and consistency in presentation:

- Please provide a clearer justification for limiting the publication period to 2023–2026. The manuscript has mentioned this period as one of the inclusion criteria, but the rationale for selecting such a relatively short timeframe has not been adequately explained.
- Please clarify the reason for including only articles available via open access. While this criterion may facilitate access to articles, it also has the potential to introduce selection bias by excluding relevant empirical studies that are not openly available. The authors should provide a brief explanation for this decision and list it as one of the study’s limitations.
- Please clarify the inclusion criteria regarding quantitative research design. The “Methods” section states that only quantitative empirical studies were included, yet Table 2 lists several studies that used mixed methods. The authors need to explain whether mixed-methods studies can still be included as long as they have a dominant quantitative component, and then adjust the inclusion criteria to ensure consistency with the articles analyzed.

Are the rationale for, and objectives of, the Systematic Review clearly stated?

Yes

Is the statistical analysis and its interpretation appropriate?

Not applicable

If this is a Living Systematic Review, is the ‘living’ method appropriate and is the search schedule clearly defined and justified? (‘Living Systematic Review’ or a variation of this term should be included in the title.)

Not applicable

Are sufficient details of the methods and analysis provided to allow replication by others?

Partly

Are the conclusions drawn adequately supported by the results presented in the review?

Yes

Reviewer Expertise:

Public Administration, Policy, and Governance

I confirm that I have read this submission and believe that I have an appropriate level of expertise to confirm that it is of an acceptable scientific standard, however I have significant reservations, as outlined above.

# Response

**Published:** 2026-10-14

**Authors:** Joni Prasetiyanto

**Affiliations:** Universitas Brawijaya Fakultas Ilmu Administrasi, Malang, East Java, Indonesia

We thank the Reviewer for the positive evaluation and constructive feedback. We appreciate these suggestions and have revised the manuscript to improve methodological transparency and consistency in reporting.

 We have added a justification for the 2023–2026 period in the “Research Methods” section.

 We have added a justification for the restrictions on open access.

 We have clarified the inclusion criteria to state that mixed-methods studies may be included if their quantitative results are relevant to the research questions.

# Reviewer report: Reviewer response for version 1

**DOI:** 10.5256/f1000research.206951.r517454

**Published:** 2026-09-15

**Authors:** Sofwan Farisyi

**Affiliations:** 1 Trisakti University ,Institut Transportasi dan Logistik Trisakti,, West Jakarta, Jakarta, Indonesia

1. Define the review construct and conceptual boundaries more precisely.

 2. Justify the 2023-2026 (?) window and Scopus only.

 3. The methods state that only quantitative studies but table 2 includes at least one mixed methods study.

 4. Result and discussion state that most studies show positive effects. Please make more detail how many studies reported positive, negative, etc for each digital innovation and sustainability outcome.

Are the rationale for, and objectives of, the Systematic Review clearly stated?

Yes

Is the statistical analysis and its interpretation appropriate?

I cannot comment. A qualified statistician is required.

If this is a Living Systematic Review, is the ‘living’ method appropriate and is the search schedule clearly defined and justified? (‘Living Systematic Review’ or a variation of this term should be included in the title.)

Not applicable

Are sufficient details of the methods and analysis provided to allow replication by others?

Partly

Are the conclusions drawn adequately supported by the results presented in the review?

Partly

Reviewer Expertise:

I have done a Systematic Literature Review for Sustainability Reporting Determinant

I confirm that I have read this submission and believe that I have an appropriate level of expertise to confirm that it is of an acceptable scientific standard.

# Response

**Published:** 2026-10-14

**Authors:** Joni Prasetiyanto

**Affiliations:** Universitas Brawijaya Fakultas Ilmu Administrasi, Malang, East Java, Indonesia

We would like to thank the reviewers for their constructive feedback. We appreciate their suggestions and have revised the manuscript to clarify the concepts, improve the transparency of our methods, and provide a more detailed discussion of our findings. Our responses are as follows:

- We have clarified the definitions of digital innovation and corporate sustainability, as well as the conceptual scope of the study, in the Introduction section.
- We have added a justification for the 2023–2026 time period to focus the study on the most recent empirical evidence. The selection of Scopus is explained based on its relevant multidisciplinary coverage, and the limitations of using a single database have been acknowledged.
- We have adjusted the inclusion criteria to clarify that mixed-methods studies may be included if their quantitative findings are relevant and can be extracted separately. The original design classification is retained in Table 2.
- We have detailed the number of studies and the direction of the findings in the Discussion section.
